# The role of food selectivity in the association between child autistic traits and constipation

**DOI:** 10.1002/eat.23485

**Published:** 2021-02-17

**Authors:** Holly A. Harris, Nadia Micali, Henriette A. Moll, Ina van Berckelaer‐Onnes, Manon Hillegers, Pauline W. Jansen

**Affiliations:** ^1^ Erasmus Medical Center Department of Child & Adolescent Psychiatry/Psychology Rotterdam Netherlands; ^2^ Erasmus Medical Center, Generation R Study Rotterdam Netherlands; ^3^ Department of Pediatrics Gynaecology and Obstetrics, Faculty of Medicine University of Geneva Geneva Switzerland; ^4^ Department of Psychiatry, Faculty of Medicine University of Geneva Geneva Switzerland; ^5^ Great Ormond Street Institute of Child Health University College London London UK; ^6^ Erasmus Medical Center, Sophia Children's Hospital Department of General Pediatrics Rotterdam Netherlands; ^7^ Leiden University Faculty of Social and Behavioural Sciences, Clinical Child and Adolescent Studies Leiden Netherlands; ^8^ Erasmus University Rotterdam Department of Psychology, Education and Child Studies Rotterdam Netherlands

**Keywords:** autism, autistic traits, child, constipation, food selectivity, gastrointestinal symptoms, mediation, picky eating

## Abstract

**Objective:**

This study examines the association between child autistic traits and constipation symptoms, and explores whether this association is mediated by food selectivity.

**Method:**

The sample included participants (*N* = 2,818) from the population‐based birth cohort, *Generation R* (Rotterdam, the Netherlands). Parents reported their child's autistic traits at 6 years (using the Social Responsiveness Scale), food selectivity at 10 years (using the Stanford Feeding Questionnaire) and the frequency and severity of constipation symptoms they experienced at 10 years (using the ROME III functional constipation diagnostic criteria). Mediation analyses tested mediation through food selectivity in the association of autistic traits and the number of constipation symptoms, adjusting for covariates.

**Results:**

There was a positive association between parent‐reported child autistic traits and constipation symptoms (*r* = 0.08, *p* < .001). We identified a significant indirect effect of autistic traits on constipation symptoms through food selectivity (*β* = 0.008, 95% Confidence Interval: 0.002, 0.014).

**Discussion:**

This study provides empirical support for the mediating role of food selectivity in the association between autistic traits and constipation. Behavioral interventions aimed to target food selectivity and support families of children with autistic traits may bolster conventional medical and nutritional treatments to alleviate gastrointestinal symptoms like constipation.

## 1 INTRODUCTION

Autism spectrum disorder (ASD) is a neurodevelopmental condition characterized by social communication deficits, and restrictive and repetitive behavioral patterns (American Psychological Association, 2013). The worldwide prevalence of ASD is approximately 1% (Lord et al., 2020), but the condition is typically diagnosed in childhood. One in every 59 children in the US receives an ASD diagnosis by age 8 years (Baio et al., 2018). Various morbidities co‐occur with ASD, for example, gastrointestinal (GI) dysfunction affects up to 82% of children diagnosed with ASD (Leader, Tuohy, Chen, Mannion, & Gilroy, 2020). Constipation is one of the most common GI symptoms experienced by children with ASD (Ibrahim, Voigt, Katusic, Weaver, & Barbaresi, 2009), affecting individuals, their families and the healthcare system (Sparks, Cooper, Hayes, & Williams, 2018). However, it is unknown if there is an ASD‐constipation association across the spectrum of autistic traits in the general population; and what mechanisms underlie this association.

“Food selectivity” is hypothesized to play a role in the association between autistic traits and constipation (Bresnahan et al., 2015). The ritualistic tendencies, sensory sensitivities and inflexibility inherent to autistic traits may manifest in food selectivity (Zickgraf, Richard, Zucker, & Wallace, 2020), prolonged disordered eating (Saure et al., 2020) and poor diet quality typically characterized by inadequate intake of nutrient‐dense foods such as fruits and vegetables (Sharp et al., 2018). Food selectivity (or “picky”/“fussy” eating) describes a child's limited dietary repertoire and aversion to certain tastes, textures, colors, types and brands of food. While this may be a transient phase for neurotypical children, food selectivity is more prevalent, more severe and more enduring in children with ASD (Leader et al., 2020). ASD can also be comorbid with Avoidant/Restrictive Food Intake Disorder (ARFID), a feeding disorder characterized by clinically significant food selectivity which negatively impacts health and psychosocial functioning (Kambanis et al., 2020). Food selective diets which are high in simple carbohydrates and fat, and low in fiber, are hypothesized to contribute to the onset of or exacerbate GI symptoms in children with ASD (Berry et al., 2015). This article examines the association between autistic traits and constipation symptoms; and whether this association is mediated by food selectivity in a general population of school‐aged children.

## 2 METHOD

### 2.1 Study design and population

Sample demographics are shown in Table 1. The study sample is embedded in *Generation R*, a population‐based birth cohort on health and development from fetal life onwards (Jaddoe et al., 2006). All pregnant women living in Rotterdam, the Netherlands, with an expected delivery date between April 2002 and January 2006 were invited to participate. The study was approved by the Medical Ethical Committee of the Erasmus Medical Center Rotterdam and written informed consent was obtained from parents of all children. Of the participants that provided full consent for participation in the early and mid‐childhood phases of Generation R (*N* = 6,036), data of the main variables of interest were available for *N* = 2,818 (47%) children. Compared to this current analytical sample, children with missing values were more likely to be non‐Western, have a lower birthweight and IQ score at 6 years, and have a higher Body Mass Index (BMI) at 10 years; and their mothers were younger and less educated (all *p*s ≤ .013).

**TABLE 1:** Population characteristics (*N* = 2,818)

	*n*	*N* (%) or mean ± *SD*
**Child**		
Sex (% boys)	2,818	1,398 (49.6)
Birthweight (grams)	2,816	3,443.5 ± 574.7
Ethnicity	2,816	
Dutch		2062 (73.2)
Western		254 (9.0)
Non‐western		500 (17.8)
Non‐verbal IQ^a^ at 6 years	2,482	104.7 ± 14.4
Constipation at 6 years, range: 0 to 3	2,718	0.20 ± 0.50
BMI Z score at 10 years	2,715	0.14 ± 0.97
*Main variables of interest*		
Autistic traits^b^ at 6 years, range: 0 to 2	2,818	0.21 ± 0.23
Food selectivity^c^ at 10 years, range: 1 to 5	2,818	2.30 ± 0.88
Constipation^d^ at 10 years, range: 0 to 11	2,818	0.82 ± 1.34
**Mother**		
Age at inclusion (years)	2,818	32.1 ± 4.3
Educational level	2,727	
High		991 (36.3)
Mid‐high		752 (27.6)
Mid‐low		703 (25.8)
Low		281 (10.3)

Abbreviations: BMI, body mass index; IQ, intelligent quotient.

^a^ Snijders‐Oomen nonverbal IQ test.

^b^ Social Responsiveness Scale.

^c^ Picky Eating subscale on the Stanford Feeding Questionnaire.

^d^ Based on the ROME III criteria, indicates sum score based on the severity of symptoms experienced.

### 2.2 Measures

#### 2.2.1 Autistic traits

At 6 years (6.1 ± 0.4 years), parents completed the 18‐item Social Responsiveness Scale short‐form (SRS) (Constantino et al., 2003). The SRS provides a valid quantitative measure of subclinical and clinical autistic traits. Parents rated the frequency of their child's behavior related to social cognition, social communication and social mannerism from “0” (never true) to “3” (almost always true). Items were averaged and higher scores indicated greater severity of autistic traits (*α* = 0.77). Based on the authors' recommended screening cutoffs (Constantino, 2002) in population‐based settings (1.078 for boys and 1.000 for girls), *n* = 39 (1.3%) of children in the sample had elevated autistic traits (29 boys and 10 girls).

### 2.3 Food selectivity

At 10 years (9.7 ± 0.3 years), food selectivity was measured via the 4‐item “picky eating” subscale from the parent‐reported Stanford Feeding Questionnaire (Jacobi, Agras, Bryson, & Hammer, 2003). Food selectivity items were measured on a scale from “1” (never) to “5” (always) and averaged (*α* = 0.84).

### 2.4 Constipation

Constipation, also assessed at 10 years, was measured via parent‐report using 6‐items based on the ROME III functional constipation diagnostic criteria (Supporting Information) (Rasquin et al., 2006). Item responses were scored to indicate the presence and severity of constipation symptoms with “0” (No), “1” (Yes, <1/week) and “2” (Yes, ≥1/week”), except for item 1 (“*In the past 2 months did your child have a bowel movement 2/week or less?*”) which had 2 responses scored as “0” (No) or “1” (Yes). Remaining items were related to fecal inconsistence, stool retention, hard and/or painful bowel movements and large stool volume. Scores were summed.

A sensitivity analysis was performed with children who met the ROME III criteria for functional constipation. Children met the criteria if they experienced ≥2 of 6 constipation symptoms at least once per week (Rasquin et al., 2006). However, the following modifications were made: one symptom on the ROME III related to fecal impaction was excluded due to difficulty in assessment via parent‐report. Also, two separate items in the parent survey measured “painful” and “hard” bowel movements, which captures one symptom on the ROME III. This symptom was present if the child experienced either or both symptoms. Finally, Irritable Bowel Syndrome (IBS) contradicts constipation diagnosis (Rasquin et al., 2006). Parents were asked if their child has been diagnosed with IBS by a doctor (“1” [Yes] or “0” [No]). Children who met the criteria for constipation with an IBS diagnosis were planned to be excluded from the sensitivity analysis.

### 2.5 Covariates

Several possible covariates are considered in the analyses. Information on child sex and birth weight was obtained from hospital/midwife registries. Child ethnicity (categorized as Dutch, Western and Non‐western) was based on country of birth of both biological parents. Trained research assistants assessed child intelligence via a nonverbal Intelligent Quotient (IQ) test at 6 years (Tellegen, Winkel, Wijnberg‐Williams, & Laros, 2005). At 6 years, parents reported whether children had constipation symptoms present using 3 items (Tharner et al., 2015). Item responses (“1” [Yes] or “0” [No]) were summed, with higher scores indicating more constipation symptoms. Children's height and weight were measured at 10 years and converted into sex‐ and age‐adjusted BMI Z scores. Maternal age and education level were assessed by postal questionnaire during pregnancy.

### 2.6 Statistical analyses

Descriptive analyses were performed in SPSS, version 25. Pearson correlations were run to describe the correlations between the main variables of interest. To examine the indirect effect of food selectivity on the association between autistic traits and constipation symptoms, analyses were performed in Mplus, version 8.4. The mediation model was estimated using maximum likelihood estimation with robust standard errors. Little's MCAR test showed that missing values on covariates were missing completely at random (*p* = .449). Full Information Maximum Likelihood was therefore used to account for missing values on the covariates. Covariates were included in the model if they modified the magnitude of the association between autistic traits and constipation >5%.

## 3 RESULTS

**FIGURE 1:**
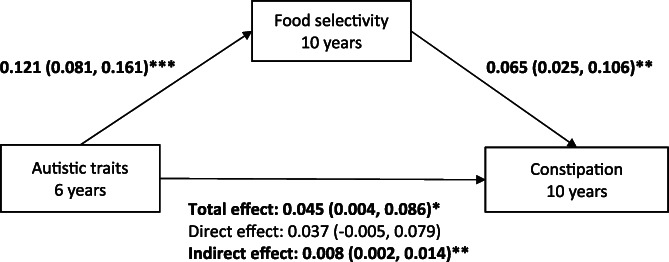
Model showing the indirect relationship between child autistic traits and constipation symptoms through food selectivity (*N* = 2,818) *p < .05, **p < .01, ***p < .001. Model adjusted for constipation at 6 years, IQ at 6 years, BMI Z score at 10 years and maternal education; Values represent the standardized coefficients (95% Confidence Intervals) for each pathway; Autistic traits assessed via the Social Responsiveness Scale; Food selectivity assessed via the Picky Eating subscale on the Stanford Feeding Questionnaire; Constipation is based on the ROME III criteria for functional constipation

Autistic traits at 6 years was positively correlated with constipation symptoms at 10 years (*r* = 0.08, *p* < .001). Autistic traits were also positively correlated with food selectivity at 10 years (*r* = 0.12, *p* < .001); as was food selectivity and constipation symptoms (*r* = 0.09, *p* < .001). Figure 1 shows the standardized path coefficients and standard errors for the mediation model of autistic traits on constipation symptoms indirectly through food selectivity, adjusting for covariates (child baseline constipation, BMI, IQ and maternal education). The indirect effect of autistic traits on constipation symptoms through food selectivity was significant, β = 0.008, 95% Confidence Interval (CI): 0.002, 0.014. Once accounting for the indirect effect of food selectivity, the direct effect between autistic traits at 6 years and constipation at 10 years was no longer statistically significant, direct effect: *β* = 0.037, 95% CI: −0.005, 0.079). The model explained 9.6% of the variance in child constipation symptoms at 10 years (*p* < .001). In the model, constipation at 6 years was the strongest predictor of constipation at 10 years, *β* = 0.284, 95% CI: 0.228, 0.341.

Of the children who met the clinical criteria for functional constipation (*n* = 86; 3.1%), only *n* = 1 child who also had an IBS diagnosis was excluded from the sensitivity analysis. An Independent samples T‐test showed that children with functional constipation had greater autistic trait scores versus those who did not meet the diagnosis (0.30 ± 0.33 vs. 0.21 ± 0.22, *p* = .015). There was no significant difference in food selectivity scores between children with and without functional constipation, although results were in the expected direction (2.48 ± 0.92 vs. 2.30 ± 0.88, *p* = .062). Therefore, mediation analysis with this binary outcome was not conducted.

## 4 DISCUSSION

The current population‐based study provides support for the link between child autistic traits at 6 years and constipation symptoms at 10 years. Importantly, findings from this study suggest that 17.8% of the total effect of autistic traits on constipation is explained by the indirect effect of food selectivity at 10 years. This confirms previous hypotheses suggesting that food selectivity could be one pathway explaining the co‐occurrence between ASD and GI issues like constipation (Bresnahan et al., 2015). Additionally, the associations explored in the current study may be indicative of an overlap between ASD and ARFID at the symptom‐level or evidence of comorbidity between the disorders. Selective eating and dietary nutritional inadequacies resulting in GI problems might be shared by ARFID and ASD.

Findings from the current study indicate the need to investigate behavior‐based selective eating interventions to alleviate or prevent constipation among children with autistic traits, together with standard medical and nutritional recommendations (Berry et al., 2015). Such interventions must be tailored to accommodate the needs of children with autistic‐like characteristics and their families. Clinicians must be alert to the role of food selectivity when treating children's constipation, and integrate an assessment of diet history (including special diets followed), preferences and family mealtime behaviors into their treatment planning. While medical nutrition therapy may address dietary factors to relieve constipation symptoms such as fiber and fluid intake, children's selective eating could hinder their adherence to dietary prescriptions, particularly when autistic traits are more severe (Berry et al., 2015). Prevention and management of constipation could be accompanied with considerations for children's behavioral responses to dietary alterations (e.g., resistance to try novel foods, tantrums and aggression), sensory processing and aversions, and provide family support.

Limitations of the current study include the use of parent‐reported measures and the lack of temporal measurement between mediator and outcome. Caution is advised when interpreting the directionality of associations, as the relationship between food selectivity and constipation may be bidirectional. For example, food selectivity could cause or exacerbate constipation symptoms, but alternatively, food selectivity could be a learned (conditioned) response to painful bowel movements (Tharner et al., 2015). Nevertheless, interventions aimed to reduce children's food selectivity and diversify nutritional intake in the long‐term could have a tenable impact on constipation management. A small percentage of the total effect of autistic traits on constipation is explained by food selectivity, suggesting that other factors are involved in the ASD‐constipation association. Only a small proportion of children met the clinical criteria for functional constipation. The relationship between food selectivity and clinically relevant functional constipation was not significant, although the results were in the expected direction. The outcome variable was based on the ROME III criteria (Rasquin et al., 2006), yet, the exact items used to assess the severity of constipation have not been validated. Furthermore, one constipation symptom was excluded from the survey as fecal impaction may be difficult for parents to report, and therefore is a limitation of the current study.

There were also many study strengths, including a large number of participants in a prospective, population‐based sample, which enabled the detection of small effects. Differences in participant characteristics between those included in the current study versus those excluded due to missing data may not be generalizable beyond socioeconomically homogenous populations. While less affluent groups were slightly underrepresented, we do not expect that associations differ between included and excluded participants. Furthermore, autistic traits were measured using a continuous indicator and therefore provided information about the broader autism profile rather than recruiting and examining children already diagnosed with ASD. Finally, while the current study shows that the effect of autistic traits on constipation was indirectly explained by food selectivity, future research is required to elucidate additional mechanisms involved. For example, food selectivity may be one of many cascading processes impacting GI symptoms in children with autistic traits.

This study provides empirical support for the mediating role of child food selectivity in the association between autistic traits and constipation. Such evidence improves the current understanding of why autistic traits may be linked to GI dysfunction. Behavioral interventions aimed to reduce food selectivity and support families of children with autistic traits may bolster conventional medical and nutritional treatments to alleviate GI symptoms.

## CONFLICT OF INTEREST

The authors declare no potential conflict of interest.

## Supporting information


**Table S1** ROME III Criteria for functional constipation and corresponding Generation R survey items.

## Data Availability

Data Availability Statement: Requests for data access can be send to datamanagementgenr@erasmusmc.nl and will be discussed in the Generation R Study Management Team.
