# Supplementary material for: The role of food selectivity in the association between child autistic traits and constipation
Source: Int J Eat Disord. 2021 Feb 17;54(6):981–5. doi: 10.1002/eat.23485 (PMC8248436; doi:10.1002/eat.23485)
Supplement: Supplementary file 1 — Table S1 ROME III Criteria for functional constipation and corresponding Generation R survey items. [file EAT-54-981-s001.docx]

**Supplementary material**

**Table S1.** ROME III Criteria for functional constipation and corresponding Generation R survey items

| **ROME III Criteria** | **Parent-reported items** | **Responses** |
| --- | --- | --- |
| ≥2 of the following symptoms for at least once per week in the 2 months prior to diagnosis… | In the past 2 months did your child… | - |
| 1. Two or fewer defecations in the toilet per week | 1. Have a bowel movement twice per week or less? | No  Yes |
| 1. At least 1 episode of fecal incontinence per week | 1. Have any stool traces in his/her underwear | No  Yes, <1/week  Yes, ≥1/week |
| 1. History of retentive posturing or excessive volitional stool retention | 1. Try to hold bowel movement in? | No  Yes, <1/week  Yes, ≥1/week |
| 1. History of painful or hard bowel movements | 1. Have any painful bowel movements?   *OR**   1. Have any hard stools (pellets)? | No  Yes, <1/week  Yes, ≥1/week |
| 1. Presence of a large fecal mass in the rectum | *Not included in survey* | |
| 1. History of large diameter stools that may obstruct the toilet. | 1. Have a large quantity of stools that could block the toilet? | No  Yes, <1/week  Yes, ≥1/week |

*Items collapsed into 1 criteria to reflect the ROME III criteria for functional constipation; meeting the ROME III criteria for functional constipation requires that at least 2 symptoms are present ≥1/week
